# The Missing Lnc: The Potential of Targeting Triple-Negative Breast Cancer and Cancer Stem Cells by Inhibiting Long Non-Coding RNAs

**DOI:** 10.3390/cells9030763

**Published:** 2020-03-20

**Authors:** Justin M Brown, Marie-Claire D Wasson, Paola Marcato

**Affiliations:** 1 Departments of Pathology, Dalhousie University, Halifax, NS B3H 4R2, Canada; justin.brown@dal.ca (J.M.B.); mc.wasson@dal.ca (M.-C.D.W.); 2 Departments of Microbiology & Immunology, Dalhousie University, Halifax, NS B3H 4R2, Canada

**Keywords:** breast cancer, long non-coding RNA, cancer stem cells, subtype, triple-negative breast cancer

## Abstract

Treatment decisions for breast cancer are based on staging and hormone receptor expression and include chemotherapies and endocrine therapy. While effective in many cases, some breast cancers are resistant to therapy, metastasize and recur, leading to eventual death. Higher percentages of tumor-initiating cancer stem cells (CSCs) may contribute to the increased aggressiveness, chemoresistance, and worse outcomes among breast cancer. This may be particularly true in triple-negative breast cancers (TNBCs) which have higher percentages of CSCs and are associated with worse outcomes. In recent years, increasing numbers of long non-coding RNAs (lncRNAs) have been identified as playing an important role in breast cancer progression and some of these have been specifically associated within the CSC populations of breast cancers. LncRNAs are non-protein-coding transcripts greater than 200 nucleotides which can have critical functions in gene expression regulation. The preclinical evidence regarding lncRNA antagonists for the treatment of cancer is promising and therefore, presents a potential novel approach for treating breast cancer and targeting therapy-resistant CSCs within these tumors. Herein, we summarize the lncRNAs that have been identified as functionally relevant in breast CSCs. Furthermore, our review of the literature and analysis of patient datasets has revealed that many of these breast CSC-associated lncRNAs are also enriched in TNBC. Together, this suggests that these lncRNAs may be playing a particularly important role in TNBC. Thus, certain breast cancer-promoting/CSC-associated lncRNAs could be targeted in the treatment of TNBCs and the CSCs within these tumors should be susceptible to anti-lncRNA therapy.

## 1. TNBCs and CSCs – in Need of New Targets

Breast cancer is a heterogeneous disease, categorized clinically based on expression of hormone receptors, estrogen receptor (ER), progesterone receptor (PR), or human epidermal growth factor 2 receptor (HER2) [1]. The presence of hormone receptors in breast tumors allows for treatment with hormone receptor antagonists in addition to chemotherapies [2]. Breast tumors lacking expression of these receptors, termed triple-negative breast cancers (TNBCs), are treated primarily with chemotherapies and are associated with worse outcomes, at least in part due to therapy resistance [3]. Transcriptome analyses have sub-classified breast cancers into at least four major subtypes (luminal A, luminal B, HER2, and basal-like) [4]. The basal-like subtype is comparatively aggressive and is enriched with TNBCs, with at least 75% of basal-like breast cancers being triple-negative [5].

The aggressiveness of TNBC/basal-like breast cancers has been attributed to the enrichment of cancer stem cell (CSC) populations within these subtypes [6,7,8,9,10,11,12,13,14]. Within tumors, CSCs are the most tumorigenic cells, initiating new tumors with high efficiency [15]. Most concerning in terms of mitigating the risk of metastasis and recurrence in the treatment of TNBC/basal-like breast cancers is the resistance of CSCs to chemotherapies [16].

Breast CSCs are typically identified by the expression of cell surface markers (CD44^high^CD24^low^ or CD133^high^), or high aldehyde dehydrogenase (ALDH^high^) activity quantified by the Aldefluor assay [15,17,18]. In addition, pluripotent stem cell markers SRY box 2 (SOX2), octamer-binding transcription factor 4 (OCT4), and NANOG are often overexpressed in the CSCs of different cancers [19]. In further connection with TNBC, stemness factors such as SOX2 and Myc are enriched in TNBC [20,21]. In fact, the gene expression signatures of TNBCs exhibited striking similarities to those of mammary stem cells [22]. The developmental Wnt/β-catenin, Notch, and Hedgehog (Hh) pathways are also more active in both CSCs and TNBC [23,24,25].

A further shared characteristic of TNBCs and breast CSCs is epithelial-mesenchymal transition (EMT), a phenotypic change in cells and process that contributes to invasion and distant metastasis [26]. EMT-inducing transcription factors are enriched in TNBCs and mesenchymal proteins are upregulated, while epithelial proteins are down-regulated [22]. EMT confers tumor cells with CSC-like characteristics [27,28].

Novel targeted therapies based on the unique molecular characteristics and mutations associated with TNBCs are being tested, including inhibitors for poly-ADP ribose polymerase (PARP) [29], phosphoinositide 3-kinase (PI3K) [30], mitogen-activated protein kinase (MEK) [31,32,33,34], heat shock protein-90 (Hsp90) [35], histone deacetylases [36], and androgens [37]. Moreover, some evidence suggests immunotherapies may work on TNBCs [38].

Given the high abundance of CSCs within TNBC/basal-like breast cancer, novel therapies that also target CSCs may better reduce the risk of relapse and improve the outcomes of TNBC patients. Components of dysregulated CSC-enriched molecular pathways (e.g., Notch, Wnt, and Hh) may exemplify novel druggable targets for signaling antagonists [16,39,40]. Additionally, increasing evidence suggests that it may be possible to target CSCs via CSC-specific or associated non-coding RNAs [41,42,43,44].

## 2. A Role for lncRNAs in Breast Cancer

Genomic and transcriptomic analyses have demonstrated that as much as 85% of the human genome is transcribed [45]. Hence, most of the genome encodes non-coding RNA transcripts, which necessitate an expanded appreciation for their diverse biological functions [46]. Long non-coding RNAs (lncRNAs) are independently transcribed RNA species greater than 200 nucleotides that lack open reading frames (ORFs) [47]. Functionally characterized lncRNAs have been shown to act as transcriptional enhancers, transcription factor decoys, transcriptional guides, scaffolds for molecular interactions, and competitive endogenous RNAs (ceRNAs) that sponge miRNAs, proteins, and other molecules [47].

Many lncRNAs promote cancer development, metastasis, and drug resistance, and exhibit elevated tissue-specific expression in many human cancers, supporting their potential clinical relevance [48]. Expression profiles of numerous lncRNAs are associated with poor prognosis, survival, and disease recurrence in breast cancer [49,50] and may provide insight into disease management strategies. Importantly, tissue-specific lncRNA expression in breast cancer may serve as a clinically useful biomarker to differentiate between normal and tumor tissue or breast cancer subtype [51,52,53,54]. Expression of lncRNAs in breast cancer may also serve as markers, which can contribute to earlier breast cancer diagnosis and enhanced efficacy in disease treatment [52,54,55,56,57,58,59]. Targeting lncRNAs may also antagonize cancer progression, improving clinical outlook. Pre-clinical evidence has supported the potential use of lncRNA antagonists such as GapmeRs (modified antisense oligonucleotides) in breast cancer treatment [60]. Hence, there are plausible therapeutic strategies that could target breast CSC-associated lncRNAs if these lncRNAs are indeed shown to be critical for promoting CSC properties and disease progression.

## 3. Breast CSC-Associated lncRNAs

**Figure 1:**
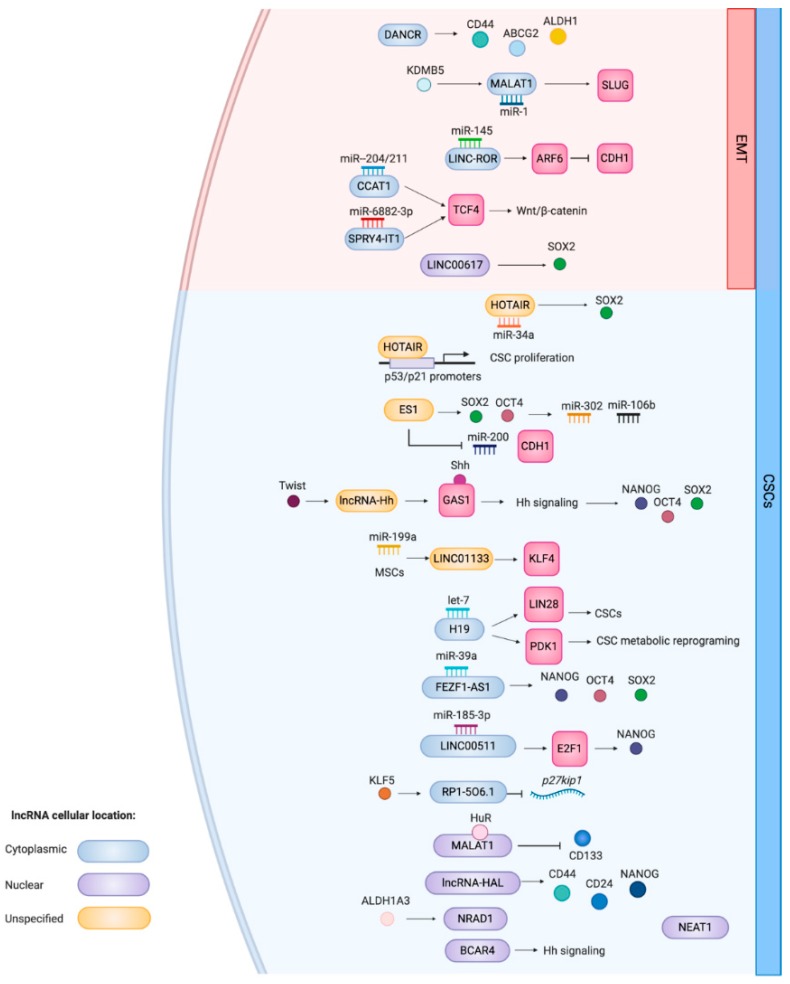
Schematic overview of the CSC and EMT-related functions of lncRNAs that have been associated with breast CSCs. This is a representation of functions summarized in Table 1. LncRNAs implicated with EMT genes and the processes are located in the red area of the cell. If known, the cellular localization of the lncRNA is indicated by the color coding. The figure was created with Biorender.com.

A literature review identified 18 lncRNAs reported to be associated with breast CSC populations and/or promoting features associated with CSCs. Some of these lncRNAs are well-studied (e.g., HOTAIR and H19), while others are more recently characterized (e.g., NRAD1). We describe the associations of these lncRNAs with breast CSC populations and summarize the findings in Table 1 and Figure 1.

### 3.1. HOTAIR

LncRNA HOTAIR (HOX Antisense Intergenic RNA) is developmentally essential by regulating expression of genes in the *HOX* family [83] and has been functionally linked to cancer progression [84,85]. HOTAIR’s pro-oncogenic activity is mediated in part by its interaction with the polycomb repressive complex 2 (PRC2). Specifically, for breast CSCs, HOTAIR regulates proliferation, self-renewal, and the tumor-forming capacity of CSCs in TNBC MDA-MB-231 cells and ER+ MCF7 cells, where it acts as a ceRNA to sponge miR-34a, allowing the upregulation of its target stemness gene, SOX2 [61]. Furthermore, by binding the promoters of tumor suppressors p53 and p21, HOTAIR affects the proliferation of the CSC populations within the breast cancer cell lines [61].

### 3.2. H19

Another developmentally important lncRNA, H19, promotes cancer progression in several cancers including breast [52,86,87,88] and is enriched in the ALDH^high^ breast CSC populations of TNBCs [63]. H19 sponges miRNA tumor suppressor, let-7, facilitating a concomitant increase in the breast CSC-enriched pluripotency factor LIN28. The sponging of let-7 by H19 also leads to the increased expression of the glycolytic enzyme pyruvate dehydrogenase kinase 1 (PDK1), which promotes the metabolic reprogramming of breast CSCs [62].

### 3.3. NEAT1

The lncRNA nuclear paraspeckle assembly transcript 1 (NEAT1) has been identified in several human cancers and has been intimately linked to breast cancer progression by stimulating cell proliferation, EMT, and metastasis [89,90]. NEAT1 is reportedly elevated in TNBC and exerts an oncogenic function (regulation of apoptosis and cell cycle progression) in the subtype by promoting tumor growth, chemoresistance and the maintenance of breast CSCs [64]. NEAT1 knockdown in TNBC cells reduced CD44^high^CD24^low^, ALDH^high^, and SOX2^high^ CSC populations.

### 3.4. MALAT1

Metastasis associated lung adenocarcinoma transcript 1 (MALAT1) was first shown to contribute to metastasis and poor patient survival in non-small cell lung cancer (NSCLC) [91] and later in the progression of TNBC [65]. MALAT1 promotes TNBC progression and aggressiveness through its regulation by the oncogenic histone demethylase KDM5B (Lysine-specific demethylase 5B), which plays a critical role in the formation and maintenance of breast CSCs [66]. Conversely, MALAT1 may act as a suppressor of breast cancer metastasis. MALAT1 and RNA-binding protein HuR form a repressive complex that regulates expression of CSC marker CD133 [67]. This complex was absent from metastatic TNBC tumor cells and present in non-metastatic cells. The absence of the MALAT1/HuR complex promotes EMT in a CD133-dependent manner.

### 3.5. BCAR4

LncRNA breast cancer anti-estrogen resistance 4 (BCAR4) was first identified as playing an oncogenic role in breast cancer [92]. Xing and colleagues [68] illustrated the potential role of BCAR4 in breast CSCs through its role in Hh signaling.

### 3.6. DANCR

Differentiation antagonizing non-protein coding RNA (DANCR) knockdown resulted in the downregulation of stemness factors CD44, ABCG2, and ALDH1 in MDA-MB-231 cells [69]. DANCR expression was correlated with TNM stages, histological grade and lymph node metastasis, and decreased survival in TNBC patients. DANCR knockdown in MDA-MB-231 and MDA-MB-468 cells reduced EMT, stemness, and inflammation [93]. Furthermore, in the normal breast epithelial cell line MCF10A, overexpression of DANCR led to acquisition of EMT, cancer stemness and inflammation properties in the normal cells [93].

### 3.7. NRAD1/LINC00284

Non-coding RNA in the aldehyde dehydrogenase 1A pathway (NRAD1), formerly referred to as LINC00284, is enriched in the ALDH^high^ CSC populations of TNBCs [70]. Its expression is regulated by CSC marker ALDH1A3. The tumorigenic NRAD1 increased mammosphere formation and contributes to gene expression regulation by ALDH1A3 through chromatin interactions.

### 3.8. LINC-ROR

Long intergenic non-coding RNA, Regulator of Reprogramming (LINC-ROR), contributes to re-programming of differentiated cells and maintenance of embryonic stem cells [94]. LINC-ROR regulates the expression of stemness factors SOX2, OCT4, and NANOG (targets of miR-145), by sponging miR-145 [95]. LINC-ROR induced EMT and invasion/migration in MCF10A cells and LINC-ROR knockdown reduced the EMT phenotype in TNBC cells [71]. Importantly, this LINC-ROR-induced EMT program produced stem-like breast cells, evidenced by the increase of the breast CSC phenotype (CD44^high^CD24^low^) in LINC-ROR-expressing MCF10A cells. Moreover, LINC-ROR-expressing MCF10A cells demonstrated enhanced mammosphere forming ability. Together, these results suggest that LINC-ROR-induced EMT promotes the formation of breast CSCs [71].

### 3.9. LINC01133

LINC01133 has been associated with the progression and suppression of multiple cancers, including breast cancer [72,96,97,98]. LINC01133 is reportedly highly enriched in TNBC relative to HER2-enriched and luminal cell lines and it is associated with poor patient survival in TNBC [72]. Mesenchymal stem cells induce expression of LINC01133 in TNBC cells, which are associated with the generation of breast CSC-like cells and modulation of the miR-199a-FOXP2 pathway [72]. LINC01133 also regulates the pluripotency factor, Kruppel-Like Factor 4 (KLF4), which promotes stemness in breast cancer [99].

### 3.10. LINC00617

LINC00617 is the human ortholog of the evolutionary conserved lncRNA TUNA, which was shown to regulate pluripotency and neural differentiation of mouse embryonic stem cells [100]. LINC00617 expression is significantly higher in breast cancer tissues relative to non-cancerous tissues, promotes the invasion/migration of MCF7 breast cancer cells and induces EMT in the TNBC MDA-MB-468 cells [73]. The LINC00617-induced EMT program stimulated an increase in the CD44^high^CD24^low^ cells, SOX2 expression, mammosphere formation potential, and metastasis. The LINC00617-mediated oncogenic activity was induced through its regulation of SOX2.

### 3.11. CCAT1

Colon cancer-associated transcript-1 (CCAT1) was first studied in colon cancer and subsequently in other cancers, including breast cancer [75,101]. In spheroid culture, breast cancer cells had a higher expression of CCAT1 [74]. CCAT1 increased expression of the stemness markers NANOG, SOX2, OCT4 and ALDH1A1, and promoted proliferation, invasion, and migration in the breast cancer cells [74]. CCAT1 is a cytoplasmic lncRNA and mediates its effects by sponging miR-204/211. Downstream targets of miR-204/211 include TCF4 (a key transcription factor in the Wnt/β-catenin signaling pathway).

### 3.12. SPRY4-IT1

SPRY4-IT1 is an intronic lncRNA transcribed from one of the introns of the SPRY4 gene, which encodes a tumor suppressor [102]. SPRY4-IT1 is upregulated in CD44^high^CD24^low^ ER+ MCF-7 cells and expression of stemness factors OCT4, c-Myc, NANOG and SOX2 are positively correlated with SPRY4-IT1 expression levels [76]. Further analysis revealed that SPRY4-IT1 mediates its stemness promoting effects by acting as a ceRNA to sequester miR-6882-3p [76], which targets TCF7L2/TCF4.Thus, SPRY4-IT1 promotes stemness by promoting the Wnt/β-catenin signaling pathway.

### 3.13. LncRNA-Hh

Thus far, Twist-induced lncRNA-Hh has been characterized only in breast cancer where it directly targets growth arrest-specific 1 (GAS1) to initiate Hh signaling in breast cancer, promoting SOX2 and OCT4 expression, EMT, tumorigenesis, and cells with CSC properties [77].

### 3.14. RP1-5O6.5

LncRNA RP1-5O6.5 is regulated by the oncogenic protein KLF5 and induced breast cancer growth and metastasis by inhibiting translation of cell cycle inhibitor p27kip1 [78]. Through its regulation of p27kip1, RP1-5O6.5 also increases stemness in breast cancer cells [78]. The proportion of CD44^high^CD24^low^ cells in TNBC cell lines with RP1-506.5 knockdown was significantly diminished.

### 3.15. LINC00511

LINC00511 has been implicated in breast cancer tumorigenesis and stemness through modulation of the miR-185-3p/E2F1/NANOG axis [79]. LINC00511 was also among a shortlist of lncRNAs identified as enriched in ALDH^high^ CSC populations of TNBCs [70]. Cytoplasmic LINC00511 functions as a ceRNA, sequestering miR-185-3p from gaining access to its target transcription factor E2F1. Increased E2F1 upregulates NANOG, contributing to stemness in breast cancer [79]. Silencing LINC00511 reduced mammosphere formation TNBCs [79].

### 3.16. FEZF1-AS1

Known to be highly expressed in various cancers, FEZF1-AS1 is involved in cell proliferation, migration, invasion and the Warburg Effect [103]. FEZF1-AS1 increases the expression of stem cell markers NANOG, OCT4 and SOX2 in MDA-MD-231 TNBC cells by acting as ceRNA sponging miR-39a [80].

### 3.17. LncRNA ES1/LINC01108

The role of lncRNA ES1 (also called LINC01108 or ENST01108) has been studied in human embryonic stem cells [104]. Knockdown of the lncRNA in TNBC MDA-MD-231 and HER2+ SKBR3 cell lines reduced stemness factors SOX2 and OCT4 and their targets, miR-302 and miR-106b, were downregulated [81]. Further, the upregulation of lncRNA ES1 increased expression of Snail/Zeb1 and miR-106b and suppressed the expression of E-cadherin and miR-200, thereby implicating ES1 as a driver of EMT.

### 3.18. LncRNA-HAL

LncRNA-HAL is located in the intronic region in the MNT gene and its expression is correlated with expression of stemness factors such as CD44, CD24 and NANOG in breast cancer [82]. lncRNA-HAL interacts with histone proteins H2B and H1.2, suggesting that lncRNA-HAL may mediate its effects by binding and altering chromatin, leading to altered gene expression [82].

## 4. Breast CSC-Associated lncRNA Correlations with CSC Markers and Signaling Pathways in TNBC and All Breast Cancer Patient Tumors

Most of the above described associations between lncRNAs and breast CSC populations were made in cell lines. To assess the clinical relevance of the associations in breast cancer, we analyzed breast cancer patient tumor gene expression correlations using RNA seq data provided in The Cancer Genome Atlas (TCGA) in the Cell 2015 dataset via cBioPortal [105,106]. We extracted Spearman’s correlation values between the individual lncRNAs and the various CSC markers and the key players in CSC-associated signaling pathways (Table 2). Notably, there were extractable expression data for only seven of the 18 lncRNAs described above (HOTAIR, H19, NEAT1, MALAT1, BCAR4, DANCR, NRAD1/LINC00284), which unfortunately prevented us from assessing the entire panel of lncRNAs. We evaluated the correlations and the direction and strength in the CSC-enriched subtype (i.e., TNBC) compared to all breast cancer subtypes. Our analyses revealed relationships between the seven lncRNAs and some of the CSC/stemness genes. Often, the expression of different lncRNAs was correlated with different genes. Additionally, the strength of significant correlations between lncRNAs and the stemness-related genes was often amplified in TNBC patient tumor data compared to the complete dataset containing the unfiltered data for all breast cancer subtypes. This result is consistent with the hypothesized importance of CSC-enriched lncRNAs in TNBC and their role in maintaining and enhancing a stemness phenotype in this breast cancer subtype.

Some interesting correlations include the strong correlation between CSC marker ALDH1A1 and MALAT1 (Table 2). This may be due to the regulation of MALAT1 by ALDH1A1, as MALAT1 was reported to be regulated by ALDH1A1 in lung cancer [107]. Similarly, NRAD1(LINC00284) was highly correlated with ALDH1A3, and we previously reported that the lncRNA is regulated by the CSC marker [70]. Furthermore, we noted a strong relationship between NRAD1 and CD133, and NRAD1 and CD24 expression (which is counterintuitive as CD24 is downregulated in CSC populations defined by CD44^high^CD24^low^, Table 2). Notably, CD24 overexpression is involved with TNBC metastasis, advanced tumor staging and decreased survival [108]. Additionally, CD133 and CD24 overexpression have been reported to promote EMT [67,108], and while our analysis did not reveal any correlation between NRAD1 (LINC00284) and EMT transcription factor expression, this finding may suggest an unreported putative function of NRAD1 in EMT.

CD49f is a marker associated with chemoresistant and tumor-initiating cell populations in TNBC [109]. We identified that NEAT1 expression is correlated with CD49f (Table 2). While the relationship between NEAT1 and CD49f has not been studied previously, this correlation may provide insight into the mechanism underlying the chemoresistance found in NEAT1-enriched tumors [64].

In patient data filtered by TNBC subtype, we observed that DANCR is positively correlated with the expression of oncogenic transcription factor c-Myc. DANCR is regulated by c-Myc in lymphoma [110], but to our knowledge, this has not been shown in breast cancer. This would be interesting to investigate given the strong correlation in patient tumors.

We also noted that H19 is highly correlated with EMT-mediating transcription factors Slug and Twist. This observation supports the role of H19 as an EMT mediator in breast cancer [111] and suggests that this function is also relevant in the TNBC subtype. The strong co-expression between MALAT1 and EMT-mediating transcription factor zinc finger E-box-binding homeobox 1 (Zeb1) further supports the clinical relevance of MALAT1 as an EMT driver through Zeb1-mediated functions. Notably, MALAT1 regulates Zeb1 expression through miRNA sponging in hepatocellular carcinoma [112]. While DANCR was found to play a role in EMT [93], our analysis of the breast cancer patient tumor data does not reflect that finding. In fact, Zeb1 and Zeb2 are negatively correlated with DANCR expression.

LncRNAs HOTAIR, H19, and MALAT1 are involved in Wnt signaling [113,114,115]. Our analysis of the breast cancer clinical data is consistent with this, as all three lncRNAs were positively correlated with Wnt signaling transcription factor TCF4.

## 5. Are Breast CSC-Associated lncRNAs Enriched in TNBCs/Basal-Like Breast Cancers?

Numerous lncRNAs are reportedly upregulated in TNBCs and contribute to their aggressiveness [49,116,117,118,119,120]. Zhang et al. performed a detailed analysis of patient tumor RNA-seq data (TCGA) and revealed more than 50 lncRNAs that are upregulated in TNBCs/basal-like breast cancers; most of which are uncharacterized and represent potentially untapped targets in TNBC [121]. Of these, approximately a fourth were later identified to be enriched in the ALDH^high^ CSC populations of TNBCs (e.g., LINC00511, NRAD1/LINC00284) [70]. Conversely, we noted that of the 18 lncRNAs thus far described to be associated with breast CSCs, 11 have been reported to be enriched in TNBCs (Table 1). This proportion is higher than expected, suggesting that breast CSC-associated lncRNAs tend to be enriched in TNBC, and this is possibly due to the enrichment of CSCs within this subtype [6,7,8,9,10,11,12,13,14].

We performed our own subtype enrichment analysis of the seven breast CSC-associated lncRNAs for which we could extract expression data from the 2015 Cell breast cancer dataset accessible at TCGA via cbioportal (Table 3). This revealed that two of the seven CSC-lncRNAs, NRAD1/LINC00284 and DANCR, are significantly enriched in TNBC/basal-like breast cancers. The striking enrichment of NRAD1 in basal-like breast cancer and TNBC suggests that NRAD1 may serve as an attractive diagnostic or prognostic target. In agreement with previous reports [122,123], our analysis also showed that NEAT1 and MALAT1 were enriched in ER+ breast cancers. This conflicts with other studies that have shown that these lncRNAs are TNBC-enriched [64,67].

For a comparison with potential protein-coding targets, we extended our analysis to include oncogene c-Myc, the hormone receptors, CSC markers, and key CSC-associated signaling pathway players (Table 3). As expected, the hormone receptors are enriched in the corresponding subtypes, and some of the CSC markers and c-Myc are enriched in TNBC/basal-like breast cancers. This data supports further study into the potential clinical relevance of at least a couple of these breast CSC-associated lncRNAs (i.e., NRAD/LINC00284 and DANCR) alongside potential protein targets, in the treatment of TNBC and basal-like breast cancers.

## 6. Clinical Value of Breast CSC-Associated lncRNAs in TNBC

Major barriers to effective remediation of TNBCs include their lack of therapeutic targets and their enrichment in tumor-initiating CSCs which confer therapeutic resistance and disease recurrence. Thus, including adjuvant therapies that target CSCs in TNBC may result in significant improvements in patient outcomes. Below, we discuss the evidence for targeting breast CSC-associated lncRNAs in the treatment of TNBC.

### 6.1. Therapeutic Targets

The investigation of lncRNAs as novel therapeutic targets provokes thoughtful questions regarding their effectiveness over protein targets. Besides the enrichment of TNBC/basal-like breast cancers (Table 3), another indicator of potential clinical value is correlation with patient outcomes. We therefore utilized KMPlotter and compared the hazard ratios of breast cancer patients with tumors with high expression of the breast CSC-associated lncRNAs or protein targets versus those with tumors that had low expression (Table 4). For the lncRNAs, we could only compare the six lncRNAs for which there was expression data. Among the lncRNAs, NRAD1 and DANCR were significantly associated with higher hazard ratios in basal-like breast cancers but not TNBC patients. This could be due to the smaller number of patients within the TNBC cohort (i.e., 40 patients). In comparison to some protein targets, we noted that ALDH1A3 was significantly associated with worse outcomes in basal-like breast cancers, as has been reported previously [124]. Notably, DANCR and NRAD1 were associated with higher hazard ratios than other potential protein targets assessed (e.g., CSC markers, c-Myc). Together, these analyses further flag NRAD1 and DANCR as candidates for further investigation as therapeutic targets.

Several pre-clinical methods have been described for targeting ncRNAs including antisense oligonucleotides (ASOs), CRISPR-Cas9 and RNAi-based methods. ASOs are DNA oligos that suppress RNA molecules by binding to them in an antisense fashion, generating a DNA-RNA duplex that can be recognized and degraded by RNase H, thereby suppressing or modifying protein expression [60,126]. ASOs have demonstrated success in both animal models and human clinical trials, and strikingly, ASOs were recently FDA-approved for clinical use for neurodegenerative diseases [60]. Synthetic and highly stable ASOs called locked nucleic acid (LNA) GapmeRs have also demonstrated some success inhibiting lncRNAs in vivo [127,128,129].

Targeting ncRNAs with CRISPR/Cas9 has been reported in cancer [130]. Specifically, lncRNA UCA1 (urothelial carcinoma-associated 1), upregulated in bladder cancer, could be inhibited by transcript-specific CRISPR/Cas9-associated gRNAs (guide RNAs) in vitro and in vivo, demonstrating its potential therapeutic value [131]. These findings suggest that there are strategies in development that will enable the clinical targeting of functionally characterized oncogenic lncRNAs that are specific to certain breast cancer subtypes (e.g., TNBC/basal-like breast cancer) and the CSCs within these tumors.

Vaidya et al. describe an effective TNBC therapy that uses nanoparticle-mediated transfer of RNAi molecules targeting the TNBC/CSC enriched lncRNA DANCR [132]. This therapy led to reduced tumor progression and was associated with no side effects in a murine xenograft model of TNBC. These results prompt the further investigation of nanoparticle-mediated targeting of oncogenic lncRNAs as a precision approach to TNBC therapy.

### 6.2. Challenges

While the lncRNA silencing methods are promising therapeutic strategies, a thorough assessment of off-target effects, potential toxicity, and drug delivery/precision targeting is required. Additionally, the multiple transcript variants of lncRNAs is more of a potential confounding factor than when considering strategies for targeting proteins. Depending on the specifics of the lncRNA silencing strategy, not all transcript variants will be targeted, possibly reducing the efficacy of the treatment if that variant is functional. In addition, the functions of most lncRNAs remain uncharacterized, further complicating the design of effective treatments and delivery strategies. Finally, whether lncRNAs represent more suitable targets over protein-coding genes is unknown and would require comparative analyses in clinical trials.

### 6.3. Biomarkers

Several studies have supported the use of lncRNAs as clinically relevant biomarkers in breast cancer. The expression of these lncRNAs may serve as valuable prognostic or diagnostic indicators in addition to markers of chemoresistance or response to treatment. Thus, relevant lncRNA expression may inform patient stratification efforts to identify those individuals who will respond best to a given treatment. The characterization of subtype-specific lncRNAs is particularly important in the context of breast cancer, where the analysis of expression levels may signify a specific subtype and aid treatment decisions.

For example, Jiang and colleagues developed an mRNA-lncRNA signature that was capable of TNBC patient classification into groups with low or high risks of disease recurrence [133]. In addition, the signature could predict response to taxane-based chemotherapy in TNBC patients. Moreover, Lv and colleagues identified a four-lncRNA signature (RP11-434D9.1, LINC00052, BC016831, and IGKV) to differentiate TNBC from non-TNBC [54]. Some other studies also identified lncRNA signatures indicative of survival [57], response to chemotherapy [59], and risk of recurrence [134].

Cancer detection through liquid biopsy via analysis of the molecules in the plasma, serum, and urine would allow for real-time, non-invasive detection of disease and promote early management strategies. Thus far, the majority of ncRNAs implicated as cancer biomarkers in liquid biopsies are miRNAs. Indeed, circulating exosomal miRNAs have been implicated as cancer biomarkers in TNBC, particularly in the blood [135].

Several lncRNAs have been detected via liquid biopsy and may serve as prognostic or diagnostic biomarkers in numerous malignancies. Of the lncRNAs discussed here, MALAT1 (in prostate cancer and multiple myeloma), HOTAIR (in NSCLC and multiple myeloma), and SPRY4-IT1 (in esophageal squamous-cell carcinoma) have been implicated as relevant biomarkers in liquid biopsies [136]. LncRNAs as liquid biopsy biomarkers have yet to be discovered for breast cancer, although this will likely come in the future.

## 7. Conclusions

Here, we have reviewed a series of oncogenic breast CSC-associated lncRNAs involved in breast cancer progression. We have also provided a review of the literature and analysis of patient data to determine the correlations of these lncRNAs with CSC markers and key signaling pathway players, subtype and patient survival. Intriguingly, our analyses unveiled correlations that have not been previously identified in the literature and may provide a starting point for further investigation of the molecular mechanisms underlying clinically pertinent links between lncRNAs and stemness-related gene expression.

LncRNAs greatly outnumber protein-coding genes in the human genome. Recently, a far-reaching analysis of lncRNAs in cancer uncovered 7941 lncRNAs that were cancer and/or lineage specific [137], further supporting the potential relevance of lncRNAs in cancer management. Due to their tissue-specific expression and upregulated expression in cancers, lncRNAs represent attractive targets and biomarkers for breast cancer diagnosis/prognosis, response to therapy, disease recurrence, and differentiation between tumor and non-tumor tissue. In addition, subtype-specific lncRNAs can help inform diagnoses and subsequent treatment routes, aiding in patient stratification efforts.

LncRNAs are involved in several aspects of breast CSCs; thus, the targeting of CSC-associated lncRNAs may represent a novel Achilles heel in the treatment of CSC-enriched TNBCs. Clinical trials for TNBCare focused on protein targets, including immune checkpoint inhibitors. Non-coding RNAs are under investigation but this is limited to miRNA. As we have demonstrated in this manuscript, numerous lncRNAs exert their regulatory oncogenic functions through interactions with miRNAs, and therefore, lncRNAs may represent equally valuable clinical targets. The tissue-specificity and enrichment of some oncogenic lncRNAs in breast CSCs and TNBCs (e.g., DANCR and NRAD1) make potential clinical investigation a possibility. A thorough analysis of the function of these lncRNAs, and characterization of the transcript variants and the potential off-target effects of the targeting strategy are first required before clinical trials are even considered.

## Figures and Tables

**Table 1:** Summary of long non-coding RNA (lncRNA) functions in breast cancer stem cells (CSCs).

lncRNA	Mechanism of Action in Breast CSCs	Evidence for TNBC Enrichment	miRNA Sponged
HOTAIR	Regulates stemness in triple-negative breast cancer (TNBC) by sponging miR-34a, promoting upregulation of SOX2. HOTAIR promotes proliferation of CSCs within breast cancer cell lines by binding the promoters of p53 and p21 [61].	-	miR-34a
H19	Sponges miRNA tumor suppressor, let-7, promoting an increase in breast CSC-enriched pluripotency factor LIN28 and glycolytic enzyme PDK1 [62].	[63]	miR-200b/c let-7b
NEAT1	Promotes tumor growth, chemoresistance and maintenance of CD44^high^CD24^low^, ALDH^high^, and SOX2^high^ CSC populations in TNBC [64].	[64]	**-**
MALAT1	Competing endogenous RNA (ceRNA) of miR-1 allowing MALAT1 interaction with Slug, prompting TNBC progression [65]. Also promotes TNBC progression by regulating KDM5B which regulates formation/maintenance of breast CSCs [66]. Binds RPB HuR, forming a repressive complex regulating CD133 [67].	[67]	miR-1
BCAR4	Regulates non-canonical Hh cascade gene transcription in a GLI2-dependent manner to promote metastasis in TNBC [68].	-	
DANCR	Involved in positive regulation of stemness factors CD44, ABCG2, and ALDH1 [69].	[69]	−
NRAD1 (LINC00284)	Oncogenic chromatin-binding lncRNA regulated by ALDH1A3 that contributes to ALDH1A3-mediated gene expression [70].	[70]	-
LINC-ROR	Induces epithelial-mesenchymal transition (EMT) to promote the formation of breast CSCs [71].	-	miR-145
LINC01133	Induced by mesenchymal stem cells (MSCs) in TNBC cells and associated with generation of breast CSC-like cells and modulation of the miR-199a-FOXP2 pathway. Regulates pluripotency factor, KLF4, which promotes stemness [72].	[72]	miR-199a
LINC00617	Induces EMT in TNBC cell lines promoting an increase in CD44+/CD24- cells, increased mammosphere formation, and metastasis through regulation of the SOX2 stemness factor [73].	-	**-**
CCAT1	Regulates stem factors NANOG, SOX2, OCT4 and ALDH1A1; Acts as a ceRNA for miR-204/211 which targets TCF4, a transcription factor in the Wnt/β-catenin pathway [74].	[75]	miR-204/211
SPRY4-IT1	Promotes stemness by sequestering miR-6882-3p which targets TCF4, allowing initiation of canonical Wnt signaling [76].	[76]	miR-6882-3p
lncRNA-Hh	Twist-induced lncRNA that directly targets GAS1 to initiate Hh signaling in breast cancer, promoting SOX2 and OCT4 expression, EMT, tumorigenesis, and cells with CSC properties [77].	-	**-**
RP1-5O6.5	Regulated by KLF5, induces breast cancer growth and metastasis by inhibiting translation of cell cycle inhibitor p27kip1, promoting stemness [78].	[78]	**-**
LINC00511	Functions as a ceRNA, sequestering miR-185-3p to upregulate NANOG via E2F1 [79]. Also, among several lncRNAs enriched in Aldefluor+ CSC populations in TNBCs [70].	[70]	miR-185-3p
FEZF1-AS1	Acts as a ceRNA for miR-39a, which targets NANOG, OCT4 and SOX2 [80].	-	miR-39a
LncRNA ES1 (LINC01108)	Acts as a ceRNA for miR-106b to decrease expression of E-cadherin and miR-200; Regulates stemness factors SOX2 and OCT4 and their downstream targets, miR-306, miR-106b [81].	-	miR-302 miR-106b miR-200
LncRNA-HAL	Regulates the expression of CD44, CD24 and NANOG [82].	[82]	**-**

**Table 2:** LncRNA co-expression with CSC markers, stemness factors, EMT genes, and players in CSC-associated signaling pathways.

Gene Names			HOTAIR	H19	NEAT1	MALAT1	BCAR4	DANCR	NRAD1
**CSC Markers**	**ALDH1A1**	TNBC	0.105	0.061	0.208	**0.328** **	−0.176	−0.113	−0.122
		All BC	0.082 *	**0.3** ****	0.066	0.077 *	−0.084 *	−0.084 *	0.128 ***
	**ALDH1A3**	TNBC	0.077	0.098	0.026	0.125	−0.021	−0.014	**0.238** *
		All BC	0.174 ****	0.189 ****	−0.124 ***	−0.113 **	−0.022	−0.009	**0.389** ****
	**CD24**	TNBC	0.013	−0.037	0.144	0.1	0.176	0.122	0.364 ***
		All BC	0.236 ****	−0.117 ***	−0.157 ****	−0.068	0.131 ***	0.092 **	0.202 ****
	**CD44**	TNBC	−0.095	−0.103	−0.28*	−0.208	−0.074	0.007	−0.019
		All BC	−0.087 *	−0.105 **	−0.069 *	−0.015	−0.106 **	0.068	0.071*
	**CD49f**	TNBC	−0.035	−0.02	−0.361 ***	−0.107	−0.141	−0.023	0.07
		All BC	−0.066	0.021	−0.173 ****	−0.156 ****	−0.132 ***	−0.067	0.097 **
	**CD133**	TNBC	−0.099	0.022	−0.033	−0.002	**0.261** *	0.051	**0.438** ****
		All BC	**0.212** ****	0.012	−0.21 ****	−0.174 ****	0.058	0.165 ****	**0.548** ****
**Stemness Factors**	**OCT4**	TNBC	−0.091	−0.192	0.045	−0.004	−0.001	−0.039	0.029
		All BC	0.11 **	−0.024	−0.14 ****	−0.112 **	0.022	0.123 ***	**0.274** ****
	**SOX2**	TNBC	0.155	−0.02	−0.061	0.085	−0.16	−0.046	−0.249 *
		All BC	0.194****	0.024	0.021	0.047	−0.019	−0.067	−0.057
	**NANOG**	TNBC	0.053	0.103	−0.131	−0.203	0.047	−0.196	0.052
		All BC	0.026	−0.017	0.039	0.019	0.037	0.086*	**0.232** ****
	**C−MYC**	TNBC	−0.317 **	−0.096	−0.084	−0.234 *	−0.036	**0.341** **	0.119
		All BC	−0.033	−0.041	−0.079 *	−0.068	0.066	**0.313** ****	0.186 ****
	**KLF5**	TNBC	−0.025	−0.002	−0.109	−0.105	−0.212	−0.128	**0.263** *
		All BC	0.169 ****	0.075 *	−0.245 ****	−0.139 ****	−0.012	0.037	**0.384** ****
	**FOXP2**	TNBC	0.134	0.026	−0.094	−0.046	−0.176	−0.059	0.054
		All BC	0.077 *	**0.225** ****	−0.039	0.05	−0.1 **	−0.114 **	0.143 ****
**EMT Transcription Factors**	**SNAIL**	TNBC	−0.07	0.083	0.136	−0.01	0.049	0.05	0.179
		All BC	0.13 ***	0.231 ****	−0.191 ****	−0.156 ****	0.134 ***	0.004	**0.263** ****
	**SLUG**	TNBC	0.034	**0.332** **	0.097	0.061	−0.023	−0.117	0.063
		All BC	0.12 ***	**0.37** ****	−0.056	−0.03	−0.06	−0.123 ***	0.194 ****
	**TWIST**	TNBC	0.011	**0.255** *	0.082	0.078	−0.02	0.014	0.04
		All BC	**0.214** ****	**0.39** ****	0.001	0.018	0.003	0.019	0.161 ****
	**ZEB1**	TNBC	**0.227** *	0.194	0.153	0.329 **	−0.037	−0.252 *	−0.051
		All BC	0.065	**0.371** ****	0.131 ***	0.184 ****	−0.102 **	−0.28 ****	0.022
	**ZEB2**	TNBC	0.027	0.072	**0.241** *	**0.244** *	−0.091	−0.232 *	−0.055
		All BC	0.021	**0.257** ****	0.025	0.042	−0.09 *	−0.268 ****	0.132 ***
**Signaling Pathways**	**STAT3**	TNBC	−0.105	−0.227 *	0.018	−0.007	−0.255 *	−0.244 *	−0.027
		All BC	−0.044	−0.05	0.156 ****	0.011	−0.125 ***	−0.207 ****	0.059
	**TCF4**	TNBC	**0.24** *	**0.34** **	0.044	**0.236** *	0.079	−0.339 **	−0.098
		All BC	0.125 ***	**0.378** ****	0.064	0.101 **	−0.078*	−0.237 ****	0.098 **

Spearman’s correlation values for the co-expression of seven lncRNAs and 19 stemness-related genes. The top value corresponds to the correlation value between the lncRNA expression and stemness gene expression in patients with TNBC while the bottom value represents Spearman’s correlation derived from data for all patients in the dataset (i.e., not discriminated by breast cancer subtype). Co-expression values highlighted in bolding indicate significant and strong co-expressions in the expected direction. Expression values were obtained from RNA Seq V2 RSEM found in the TCGA Cell 2015 dataset retrieved using the cBioPortal online software [105,106]. * = *p*-value < 0.05, ** = *p*-value < 0.01, *** = *p*-value < 0.001, **** = *p*-value < 0.0001.

**Table 3:** LncRNA and protein target fold-enrichment by breast cancer subtype.

Targets		Subtype						
		Basal	Luminal A	Luminal B	HER2+	TNBC	ER+	PR+
**lncRNA**	HOTAIR	0.84	1.01	0.94	1.12	1.09	0.96	0.96
	H19	1.04	0.87	0.68 *	0.84	1.36	0.97	0.97
	NEAT1	0.46 ****	1.15 *	1.01	1.01	0.46 ****	1.13 **	1.17 ****
	MALAT1	0.56 ****	1.09	1.17	0.9	0.58 ****	1.1	1.11 ****
	BCAR4	0.83	0.83	0.88	1.52	0.40 *	1.15	0.66
	DANCR	1.63 ****	0.81 ****	0.98	1.03	1.43 **	0.89 **	0.88 ****
	NRAD1 (LINC00284)	5.38 ***	0.24 ****	0.16 ****	0.81	4.09 *	0.26 ****	0.24 ****
**Protein Target**	ER (ESR1)	0.03 ****	1.42 ****	1.38 ****	0.70 ***	0.04 ****	1.25 ****	1.29 ****
	PR (PGR)	0.01 ****	1.48 ***	0.92	0.52 ****	0.08 ****	1.26 *	1.43 ****
	HER2 (ERBB2)	0.38 ****	0.61 ***	1.09	3.58 ****	0.22 ****	0.88	0.80 **
	MYC	1.79 ****	0.73 ****	0.84 *	0.72 ****	1.69 ****	0.89 *	0.87 ****
	ALDH1A3	1.76 **	0.78 **	0.57 ****	1.05	2.01 ***	0.76 ***	0.77 ****
	ALDH1A1	0.75	0.83 **	0.76	0.98	1	0.99	1
	CD44	1.14	1.07	0.81 *	0.77 ****	1.24	0.96	0.99
	CD133 (PROM1)	3.87 ****	0.38 ****	0.44 ****	1.01	3.71 ****	0.48 ****	0.45 ****
	β−catenin (CTNNB1)	1.11	1.02	0.88 ***	0.97	1.12	0.99	0.98
	TCF4	0.66 ****	1.10 *	0.86 **	1.02	0.81 **	1.05	1.07 ****

Fold-enrichment was calculated from mRNA expression (RNA Seq V2 RSEM) data from TCGA (Cell, 2015) dataset retrieved using the cBioPortal online software [105,106]. Fold-enrichment represents the mean gene expression for patients with breast cancers of a particular subtype over the mean gene expression for all breast cancer patients (*n* = 816). The sample sizes for each subtype are as follows: Basal (*n* = 107); Luminal A (*n* = 201); Luminal B (*n* = 122); HER2 (*n* = 120); TNBC (*n* = 82); ER+ (*n* = 594); PR+ (*n* = 521). *p*-values represent the difference between the mean of the two groups, calculated with a t-test using R v3.6.1. * = *p*-value < 0.05, ** = *p*-value < 0.01, *** = *p*-value < 0.001, **** = *p*-value < 0.0001.

**Table 4:** LncRNA hazard ratio by breast cancer subtype.

Targets		Subtype							
		Basal	Luminal A	Luminal B	HER2	TNBC	ER+	PR+	All Breast Cancers
**lncRNA**	HOTAIR	1.04	1.30 *	0.96	1.36	0.85	1.35 *	1.43	1.15
	NEAT1	0.69 *	0.63 **	0.53 ****	0.66	1.27	1.16	1.05	0.51 ****
	MALAT1	0.83	0.98	1.03	1.11	0.62	1.04	1.09	0.83 *
	BCAR4	0.98	0.99	0.77	1.11	1.01	1.04	0.94	0.94
	DANCR	1.60 **	0.83	0.80	0.73	1.21	0.79	0.65 *	0.94
	NRAD1 (LINC00284)	1.66 **	0.81	0.9	1.12	1.05	0.87	0.8	1.06
	LINC01133	1.05	0.94	1.26	1.00	0.87	1.01	1.19	1.24 **
**Protein Target**	ER (ESR1)	0.81	0.85	0.92	0.62 *	0.91	0.94	1.07	0.67 ****
	PR (PGR)	0.63 **	0.53 ****	0.77	0.86	0.94	0.56 ****	0.72	0.53 ****
	HER2 (ERBB2)	0.85	0.77 **	1.1	1.05	0.91	1.09	0.93	0.93
	c-MYC	1.02	1.06	0.94	0.82	0.81	1	1.06	1.12 *
	ALDH1A3	1.41 **	0.89	0.95	0.99	1.18	0.93	0.81	1.04
	ALDH1A1	0.67 **	0.8 **	0.87	0.68 *	0.64 *	0.86	0.97	0.74 ****
	CD44	0.97	0.67 ****	0.7 ***	1.01	0.85	0.68 ****	0.51 ***	0.72 ****
	CD133 (PROM1)	1.2	0.85	0.85	0.67 *	0.88	0.81 **	0.64 *	1.05
	Β-catenin (CTNNB1)	1.08	0.86	0.9	1.47	1.4	0.84 *	1.01	0.89 *
	TCF4 (TCF7L2)	1.15	0.87 *	1.21	1.45	1.25	0.97	0.95	1.01

Hazard ratios between high and low expression of lncRNAs were analyzed using the KMPlotter breast cancer dataset [125] divided by subtype. The sample sizes for each subtype are as follows: Basal (*n* = 360), Luminal A (*n* = 841), Luminal B (*n* = 407), HER2 (*n* = 156), TNBC (*n* = 40), ER+ (762), PR+ (*n* = 489), all subtypes (*n* = 1764). * = *p*-value < 0.05, ** = *p*-value < 0.01, *** = *p*-value < 0.001, **** = *p*-value < 0.0001.
